# Efficacy and safety of lemborexant vs placebo in treating adults with insomnia disorder: a systematic review and meta-analysis of 1976 patients

**DOI:** 10.1007/s00210-025-04072-4

**Published:** 2025-04-17

**Authors:** Mohamed A. Alsaied, Abdelrahman M. Elettreby, Ibrahim Serag, Jaisingh Rajput, Nourhan Abas Zabady, Huansheng Li, Ahmed A. Abo Elnaga

**Affiliations:** 1 Faculty of Medicine, Mansoura University, Mansoura, Egypt; 2 Department of Family Medicine, Baptist Family Medicine Residency Program, Vaughn Clinic, Montgomery, AL USA; 3 Faculty of Science, Damanhour University, Damanhour, Egypt; 4 Zhejiang Institute of Economics and Trade, Hangzhou City, Zhejiang Province China

**Keywords:** Dual orexin receptor antagonist, Insomnia, Lemborexant, Sleep disorders

## Abstract

Insomnia is a typical sleep disorder in which an individual finds it difficult to fall asleep and stay asleep resulting in poor daytime functioning and decreased health and quality of life. The orexin system, which regulates wakefulness and arousal, is often overactive in individuals with insomnia, disrupting normal sleep patterns. Lemborexant, a dual orexin receptor antagonist, works through the inhibition of the orexin system, thus facilitating increased sleep onset and maintenance of sleep. This systematic review and meta-analysis seek to determine the effectiveness and safety of lemborexant in the treatment of insomnia. A comprehensive search was conducted on PubMed, Scopus, Web of Science, and Cochrane Library, from inception to September 2024. Four randomized controlled trials (RCTs) assessing the efficacy and safety of lemborexant for patients with confirmed DSM-5 diagnosis of insomnia as compared to placebo were included. By adopting a random-effect inverse variance model, RevMan was used to pool dichotomous and continuous data. We employed the ROB2 methodology to evaluate the quality of the evidence, so ensuring the reliability of the findings obtained throughout these investigations. Four studies with a total of 1976 patients were included. Lemborexant was superior to placebo in decreasing sleep onset latency and wake after sleep onset: (MD =  − 9.23 min, *P* = 0.02 and MD =  − 19.9 min, *P* < 0.0001) with 5 mg and (MD =  − 12.56 min, *P* = 0.004) and (MD =  − 22.24 min, *P* < 0.0001) with 10 mg, respectively. In addition, sleep efficiency was statistically significantly higher in the lemborexant group (MD = 6.08%, *P* < 0.0001) and (MD = 7.46%, *P* < 0.0001) with 5 mg and 10 mg, respectively. Regarding safety analysis, Treatment emerged adverse events (TEAEs) and somnolence were statistically significantly higher in the lemborexant group (RR = 1.94, *P* < 0.0001) and (RR = 4.95, *P* < 0.0001), respectively. In accordance with this systematic review and meta-analysis, lemborexant is an effective pharmacotherapy for the treatment of insomnia as it improves sleep onset latency, wake after sleep onset, and sleep efficiency. Both formulations of 5-mg and 10-mg doses were well tolerated with no significant difference in their effect; however, somnolence was more common relative to placebo. Lemborexant appears to fill a therapeutic gap in the treatment of insomnia but should be used with caution and smaller dose (5 mg) in those who are at risk of developing an excessive daytime somnolence state.

**Supplementary Information:**

The online version contains supplementary material available at 10.1007/s00210-025-04072-4.

## Introduction

According to the DSM-5, insomnia is commonly defined as dissatisfaction with sleep quantity or quality, trouble initiating or maintaining sleep, and early morning waking despite attempts to sleep with an inability to return to sleep (American Psychiatric Association 2013). It affects roughly 30% of the general population who report one or more insomnia symptoms (Roth 2007), with 6 to 10% of patients meeting the DSM-5 diagnostic criteria (Buysse 2013; Morin and Benca 2012). Insomnia is more common among women and the elderly (Xu et al. 2011; Rybarczyk et al. 2013). Sleep disorders can be a primary disorder or secondary symptom of a more serious medical or neurological issue. Patients with insomnia usually suffer from fatigue, decreased cognitive performance, and mood disruption (Qaseem et al. 2016). Multiple studies have linked insomnia to significant impairments in an individual’s quality of life, as evidenced by the decline in the short Form Health Survey of the Medical Outcomes Study (SF-36) among insomnia sufferers (McHorney et al. 1993, 1992). Furthermore, in a multicentric investigation, Katz et al. discovered that insomnia is associated with a deterioration in quality of life comparable to chronic conditions such as congestive heart failure and severe depression (Katz and McHorney 2002).

Cognitive behavioral therapy for insomnia (CBT-I), benzodiazepines, and benzodiazepine receptor agonists are some of the key approaches to the treatment of insomnia, but it is observed that around 40% of patients do not achieve long-term relief using these therapies (Morin et al. 2009). Of all therapeutic modalities available for chronic insomnia, CBT-1 is preferable as it includes sleep–wake behavioral therapy, stimulus control, sleep restriction, relaxation, and cognitive therapy (Morin et al. 2006). The efficacy of CBT-I has been shown in meta-analyses of randomized controlled trials, and it has been demonstrated to be equal to pharmacotherapy during acute treatment and more effective for long-term treatment (Smith et al. 2002). Benzodiazepines and benzodiazepine receptor agonists are widely prescribed classes of medication for insomnia, acting by strengthening the flip-flop sleep switch. However, their safety and efficacy are restricted by the development of tolerance and an elevated risk of dependency with long-term use (Ashton 2005). Several million people worldwide are dependent on these drugs, with many experiencing long-term side effects and increased morbidity and mortality (Kripke 2009). An alternative pharmacological approach is to directly block wake-promoting adrenergic and cholinergic neurotransmission. Over the past decades, the use of antihistamines and tricyclic antidepressants has increased, but they are associated with substantial side effects such as rebound insomnia after withdrawal, liver dysfunction, and heart rhythm disturbances (Buscemi et al. 2007). They may require medical monitoring and are not yet approved for long-term use in Europe.

In 2019, the Food and Drug Administration (FDA) approved the use of lemborexant, an oral dual orexin receptor antagonist, for insomnia treatment. In vitro studies showed that lemborexant works as a competitive antagonist at OXR1 and OXR2 (Yoshida et al. 2015), which disrupts orexin neurotransmission to enhance sleep onset and maintenance while maintaining the capacity to respond to external stimuli. It was observed that lemborexant exhibited rapid binding kinetics on orexin receptors (OXRs). It also displayed significant selectivity for OXR1 and OXR2 in comparison to 88 other physiologically relevant receptors, transporters, and ion channels. Furthermore, it exhibited rapid dissociation from these receptors, in contrast to other orexin receptor antagonists (such as suvorexant and daridorexant), which typically dissociate slowly (Beuckmann et al. 2017). Therefore, it is anticipated that lemborexant will facilitate the onset of sleep while simultaneously lowering the likelihood of somnolence the following day (Beuckmann et al. 2017).

This systematic review and meta-analysis of randomized clinical trials aimed to comprehensively evaluate and assess current evidence regarding efficacy and safety of lemborexant in the treatment of insomnia.

## Methods

### Protocol and registration

This systematic review and meta-analysis will be conducted using the Preferred Reporting Items for Systematic Reviews and Meta-Analyses (PRISMA) criteria (Page et al. 2021), as well as specific instructions from the Cochrane Handbook for Systematic Reviews of Interventions (Higgins et al. 2019). We established the methods before conducting the review, and no significant deviations from the protocol were noted. The review protocol has been registered with the International Prospective Register of Systematic Reviews (PROSPERO) by the number CRD42024591019.

### Eligibility criteria

#### Inclusion criteria

This systematic review and meta-analysis included studies fulfilling the following criteria:

- ○ Population: adult patients, whether elderly or not, are diagnosed with insomnia according to diagnostic and statistical manual of mental disorders fifth edition (DSM-5)
- ○ Intervention: oral lemborexant
- ○ Control: placebo
- ○ Outcomes: this study focused on efficacy outcomes and safety profile of lemborexant including the following efficacy outcomes:
  - Sleep onset latency (SOL)
  - Sleep efficiency (SE)
  - Wake after sleep onset (WASO)
    and safety profile including the following events:
  - Somnolence
  - Treatment emerged adverse events (TEAEs)
  - Treatment-related TEAEs
  - TEAEs leading to drug discontinuation
  - Headache
  - Serious TEAEs
- ○ Study design: randomized controlled trials

#### Exclusion criteria

Studies will be excluded from systematic review and meta-analysis if they fail to meet the established inclusion criteria. Observational studies, case reports, case series, and non-randomized controlled trials, will not be considered. Similarly, studies that assess interventions or comparators unrelated to the research question will be excluded. If studies lack sufficient outcome data or if data cannot be extracted or obtained from the authors, they will also be omitted from the analysis.

### Search strategy

Search strategy was formulated using relevant keywords with the help of MeSH database to find relevant Mesh terms. A comprehensive literature search was carried out on various databases including PubMed, Scopus, Web of Science, and Cochrane Central Registry of Controlled trials (CENTRAL) using the following search strategy: (lemborexant OR (lem 10)) AND (insomnia OR agrypnia OR hyposomnia OR sleeplessness). The search included English articles published up until 1st of September 2024 with no other restrictions. Additionally, gray literature, including conference proceedings and unpublished studies, will be identified using sources such as ClinicalTrials.gov and the World Health Organization International Clinical Trials Registry Platform (ICTRP). To ensure the inclusion of relevant studies, reference lists of included articles and previous reviews will be manually searched.

### Study selection and data extraction

To avoid any instances of duplication, the search results were imported into EndNote version 20 (EndNote | The Best Citation Reference Management Tool n.d.). Following that, two reviewers evaluated the remaining publications’ titles and abstracts using the Rayyan tool (Ouzzani et al. 2016) to establish their appropriateness for the study. If necessary, a third reviewer would intervene after the original reviewers had discussed and resolved any disagreements in their judgement. Finally, two unbiased evaluators manually collected the data and reported the findings on a standardized Google sheet. Extracted data includes the following research characteristics: author, year of publication, location of the study, study design, and lemborexant dose as well as timing of administration. Participant demographics include sample size, age, sex distribution, insomnia severity index, sleep onset latency, subjective sleep efficiency, and wake after sleep onset.

### Risk of bias assessment

To assess the probability of bias in the included clinical studies, we used the Cochrane risk-of-bias tool for randomized trials (ROB 2) (Sterne et al. 2019). The tool is organized into distinct domains of bias, each addressing a different component of trial design, implementation, and documentation. Each domain employs a unique set of “signaling questions” to collect information about trial features that are relevant to the possibility of bias. An algorithm uses the responses to these questions to provide a recommended estimate of the likelihood of bias in each domain. This assessment might be classified as “low” or “high” risk of bias, or as “some concerns.” Two distinct reviewers examined each domain individually. The goal of this assessment was to get a complete understanding of the potential biases that could impact the accuracy and consistency of the research included. The ROB2 for cross over trials was implemented in assessing the cross over study.

### Statistical analysis and heterogeneity

Meta-analysis was performed using Review Manager software (RevMan v.5.4) (RevMan. n.d.) on the extracted outcome data that present in at least 3 of the included studies. Mean difference (MD) was used as the effect size for continuous outcomes with a 95% confidence interval (CI). For binary outcome data, the number of events and the total patient count were pooled to determine the risk ratio (RR) along with a 95% CI. The level of statistical significance was set to be *P* < 0.05. If SD deviation was missing, it was calculated using other statistical measures such as standard error (SE), or CI. The change from baseline values were extracted instead of final values. Data represented by figures were extracted using Plot digitizer online app (Aydin and Yassikaya 2021). The inverse variance random-effect model was adopted rather than a fixed effect model yielding a more conservative estimate of the pooled effect and generalizable results (Cochrane handbook for systematic reviews of interventions n.d.). Heterogeneity was evaluated using the chi-square test with a significance level of *P* ≤ 0.1 and an *I*^2^ value greater than 40%. We place greater emphasis on the *I*^2^ value, as it performs better than the chi-square test when the number of included studies is small. In cases of significant heterogeneity, we conducted a sensitivity analysis by removing one study in multiple scenarios. Leave one out analysis was also performed to evaluate the robustness of the results. A subgroup analysis was performed based on different doses. Finally, we could not assess publication bias using funnel plots as they are not reliable when the number of included studies is less than ten (Egger et al. 1997; Sterne and Egger 2001).

## Results

### Study selection

**Fig. 1:**
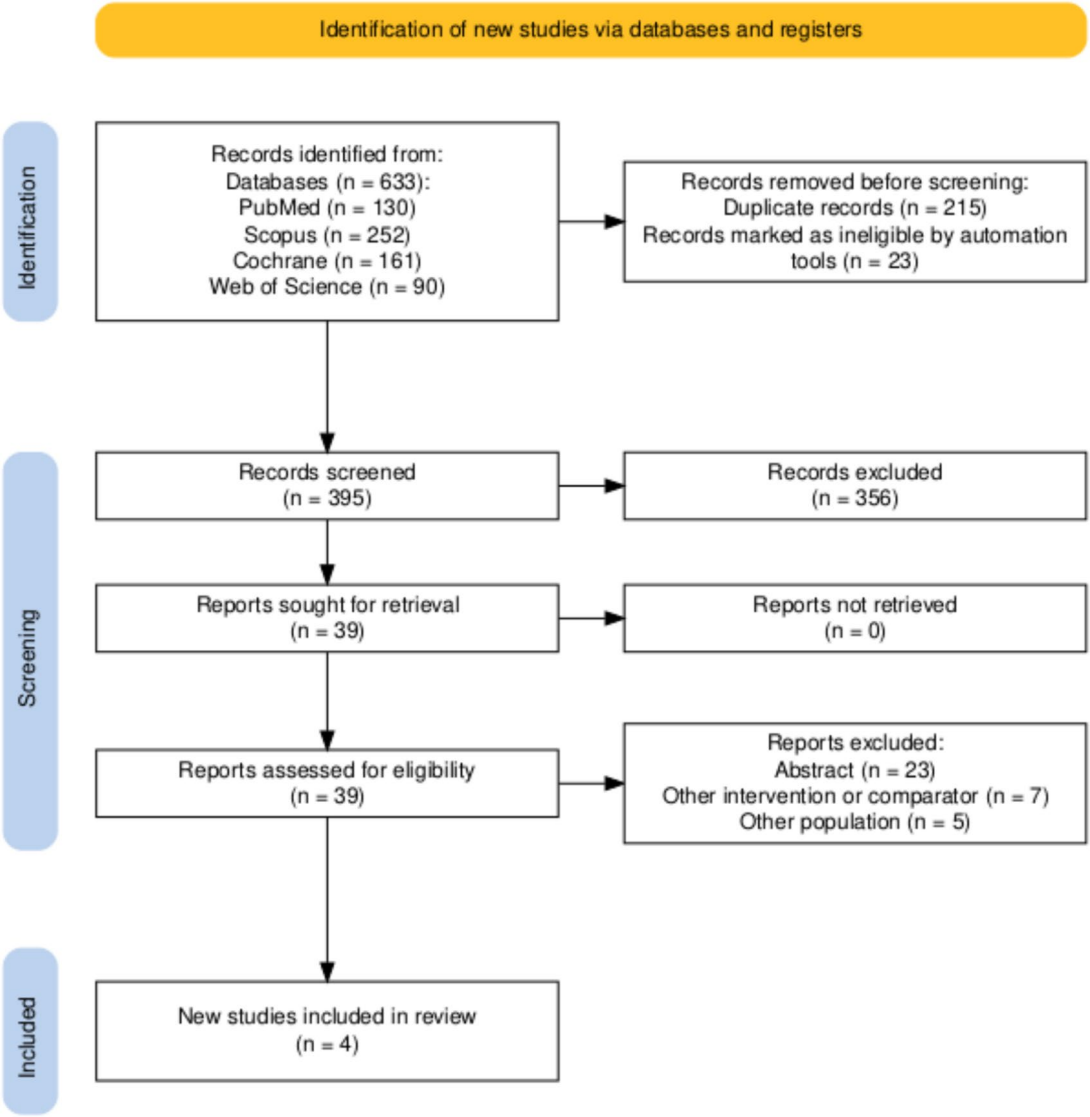
PRISMA flow diagram showing the selection process

A comprehensive electronic literature search was undertaken across multiple databases, specifically PubMed, Scopus, Web of Science, and Cochrane, yielding a cumulative total of 633 records. Following the elimination of 238 duplicate entries, a final set of 395 distinct studies remained for the purposes of title and abstract screening. The title and abstract of each one of these studies were reviewed for eligibility yielding 39 records for full-text assessment. Ultimately, 4 studies were found to meet inclusion criteria and were thus included in the systematic review and meta-analysis. The detailed flowchart of screening process is demonstrated in Fig. 1.

### Study characteristics

The selection process yielded the identification of 4 studies that met the eligibility criteria (Murphy et al. 2017; Rosenberg et al. 2019; Kärppä et al. 2020; Mayleben et al. 2021) with a total sample size of 2150 patients. This systematic review consisted of four prospective randomized clinical trials (RCTs) involving a total of 2150 patients, published between 2017 and 2021 and conducted on adult patients with insomnia disorder meeting Diagnostic and Statistical Manual of Mental Disorders, Fifth Edition (DSM-5) criteria. All included studies were multicentric conducted in different medical centers in North America (USA), Asia, and Oceania. Furthermore, all studies were phase 2 or 3 randomized, double-blind, parallel-group trials except Mayleben 2021, which was phase 1 randomized crossover study over 4 periods. The intervention was lemborexant 5 mg and 10 mg given orally within 30 min of habitual bedtime at night for a period ranged from 15 days up to 6 months. All control groups received oral placebo as a study comparator. The detailed characteristics of included studies are presented in Table 1 and baseline characteristics of patients in included studies are elicited in Table 2.

**Table 1:** Summary of included studies

Study ID	Study design	Registration number	Location	Population	Intervention	Comparator	Sample size		
							Total	Intervention	Comparator
Karppa 2020	Phase 3 RCT	NCT02952820	Multicenter (119 sites in North America (45), Europe (34), Asia (35), and Oceania (5))	Adults meeting (DSM-5) criteria of insomnia with a history of (sSOL) ≥ 30 min and/or (sWASO) ≥ 60 min at least three times a week	G1: 5 mg lemborexant G2: 10 mg lemborexant *5 min before time intended to sleep*	Placebo	971	G1: 323 G2: 323	325
Murphy 2017	Phase 2 RCT	NCT01995838	Multicentric in USA	Adults 19 to 80 years of age meeting DSM-5 criteria for insomnia with (LPS) average of ≥ 30 min with neither night < 15 min; and/or WASO average of ≥ 30 min with neither night < 20 min; and an SE average of ≤ 85% with neither night > 87.5%	Lemborexant doses (1 mg, 2.5 mg, 5 mg, 10 mg, 15 mg, or 25 mg per day) *30 min before a subject’s median habitual bedtime when in clinic and 30 min before self-selected bedtime when at home*	Placebo	291	235 for all doses	56
Rosenberg 2019	Phase 3 RCT	NCT02783729	Multicentric (at 67 sites in North America and Europe)	Women (+ 55 years) and men (+ 65 years) meeting DSM-5 criteria for insomnia with (sWASO) of 60 min or more at least 3 nights per week in the previous 4 weeks, regular time spent in bed (7–9 h), evidence of sleep maintenance insomnia, and (ISI) score of 13 or greater	G1: 5 mg lemborexant G2: 10 mg lemborexant	G3: 6.25 mg of zolpidem G4: Placebo	1006	G1: 266 G2; 269	G3: 263 G4: 208
Mayleben 2021	Phase 3 cross over RCT	NCT02350309	Two centers in USA	Adults’ meeting (DSM-5) criteria of insomnia with a history of (sSOL) ≥ 30 min and/or (sWASO) ≥ 60 min, both recorded by sleep diary on at least 3 of 7 consecutive nights during screening. and Subjects who had a regular time in bed of 7–9 h, with regular bedtime between 21:00 and 24:00 and regular waketime between 05:00 and 09:00, and (ISI) score ≥ 15	First 3 periods: lemborexant 5 mg, lemborexant 10 mg Period 4: all subjects received an open-label single oral dose of flurazepam 30 mg	69			

*DSM-5*, Diagnostic and Statistical Manual of Mental Disorders, Fifth Edition; *sSOL*, subjective Sleep onset latency; *sWASO*, subjective Wake after sleep onset; *ISI*, insomnia severity index; *SE*, sleep efficiency

**Table 2:** Baseline characteristics of included studies

Study ID	Study arms	Sample Size	Age (years), *mean (SD)*	Sex (female), *n* (%)	Duration	BMI (kg/m^2^), mean (SD)	Insomnia severity index total score, mean (SD)	Sleep onset latency (min), mean (SD)	Subjective sleep efficiency (&), mean (SD)	Wake after sleep onset (min), mean (SD)
Karppa 2020	Placebo	318	54.5 (14)	216 (67.9%)	6 months	27.2 (5.5)	19 (3.1)	56.31 (33.36)	61.34 (17.84)	132.49 (80.2)
	Lemborexant 5 mg	316	54.2 (13.7)	209 (66.1%)		27.3 (6.3)	19.6 (3.3)	54.05 (31.9)	63.14 (18.23)	132.77 (85.52)
	Lemborexant 10 mg	315	54.8 (13.7)	222 (7.5%)		27.2 (5.6)	19.1 (3.4)	58.17 (38.35)	62.03 (17.25)	136.83 (87.39)
Murphy 2017	Placebo	56	47.1 (15.6)	34 (64.3%)	15 days	26.8 (5.1)	NR	61 (32)	62.8 (13)	118.4 (56.4)
	Lemborexant 5 mg	38	51.1 (14.3)	(60.5%)		26.6 (4.1)	NR	61.9 (36.7)	66 (11.6)	102.7 (50.9)
	Lemborexant 10 mg	32	47.1 (13.7)	(62.5%)		26.3 (4.4)	NR	48.2 (27.9)	66.4 (11.8)	108.7 (37.9)
Rosenberg 2019	Placebo	208	63.4 (6.4)	184 (88.5%)	1 month	NR	19.4 (3.6)	43.9 (33.6)	68.9(9.6)	111.8(37.2)
	Zolpidem ER 6.25 mg	263	64.3 (7.1)	226 (85.9%)		NR	19.2 (3.5)	44.5 (38.3)	68.1 (11.4)	114.31 (39.9)
	Lemborexant 5 mg	266	63.7 (6.8)	229 (86.1%)		NR	18.9 (3.5)	44.9 (36.5)	68.4 (11.3)	113.4 (39)
	Lemborexant 10 mg	269	64.2 (6.9)	230 (85.5%)		NR	19 (3.3)	44.6 (33)	67.9 (10.8)	114.8 (40)
Mayleben 2021	All patients	69	50.2 (12.91)	51 (73.9%)	3 weeks	27.3 (4.39)	21.4 (3.67)	55 (42.55)	64.4 (11.95)	110 (39.73)

*NR*, not reported

### Risk of bias in studies

**Fig. 2:**
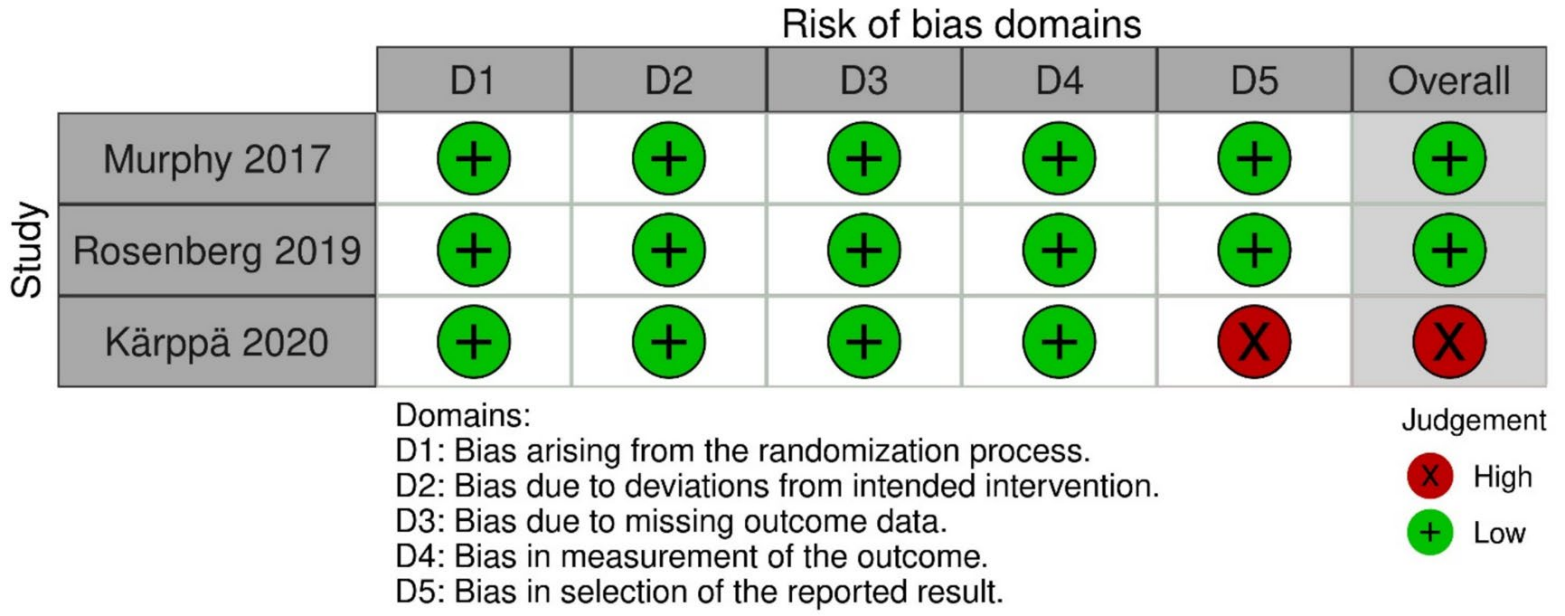
Quality assessment of parallel RCT using ROB2 tool for crossover studies

**Fig. 3:**
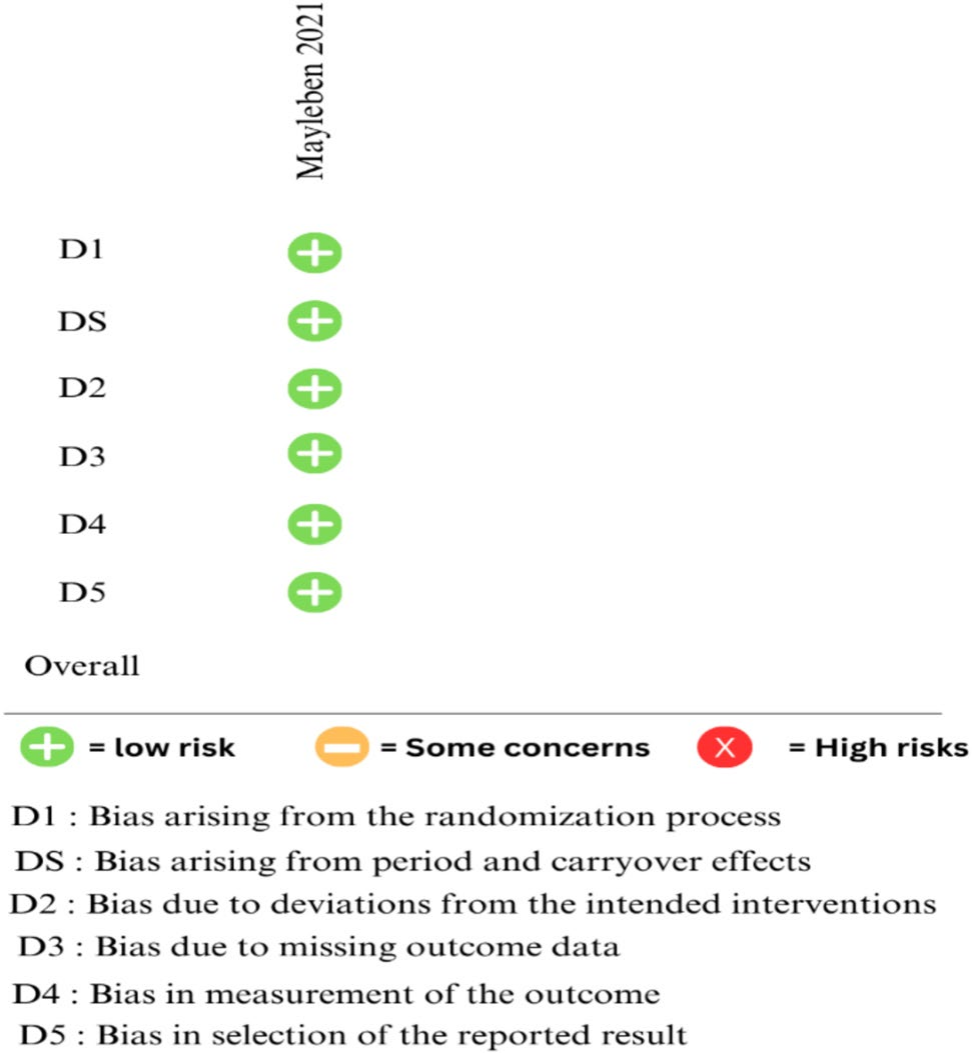
Quality assessment of crossover RCT using ROB2 tool for crossover studies

The risk of bias of included double armed parallel clinical trials was assessed using ROB 2 tool considering five main domains. The evaluation of bias for the included clinical trials revealed that two studies were at low risk for bias in all domains, while Karppa 2020 exhibited high risk in the fifth domain. Figure 2 shows detailed illustration of the risk of bias of double armed parallel clinical trials. Furthermore, risk of bias assessment of the cross over study was done using ROB2 for cross over trials tool, that revealed that Mayleben 2021 had low risk of bias in all domains (Fig. 3).

### Efficacy outcome

**Fig. 4:**
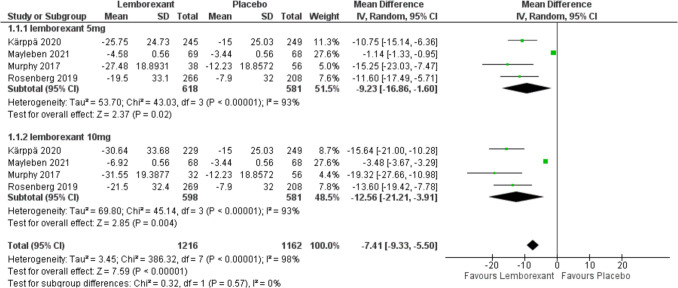
Forest plot showing the change in sleep onset latency from baseline

**Fig. 5:**
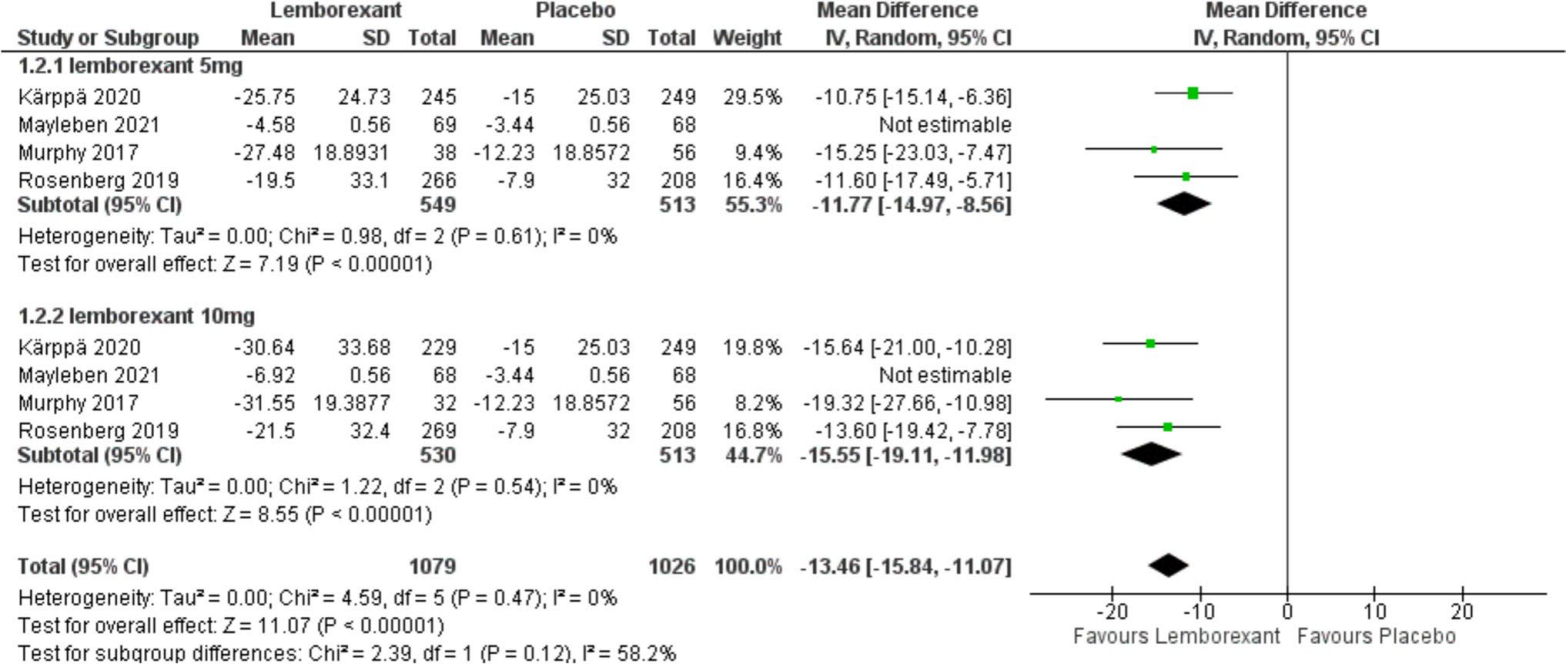
Forest plot showing sensitivity analysis for the change in sleep onset latency from baseline outcome by removing Mayleben 2021

The change in sleep onset latency from baseline was assessed in all included studies (Murphy et al. 2017; Rosenberg et al. 2019; Kärppä et al. 2020; Mayleben et al. 2021) and estimated as the time, in minutes, from the attempt to sleep until sleep onset. Mayleben 2021 had utilized an objective method for assessing the sleep onset while the others used a subjective method which was sleep diaries. A subgroup analysis was conducted based on different doses: 5 mg and 10 mg. Meta-analysis results revealed that lemborexant could significantly decrease the sleep onset latency in patients with insomnia compared to placebo group (MD =  − 9.23, 95% CI [− 16.86 to − 1.6], *P* = 0.02 and MD =  − 12.56, 95% CI [− 21.21 to − 3.91], *P* = 0.004) with 5 mg and 10 mg simultaneously. There was no statistically significant difference between the two groups (*P* = 0.57). The forest plot showed high heterogeneity {(*I*^2^ = 93%)} with 5 and 10 mg but when we removed Mayleben 2021, the pooled results of heterogeneity showed a dramatic difference (I^2^ decreased from 93 to 0%) with (*P* < 0.0001) in the meta-analysis under the random-effect model (Figs. 4 and 5).

**Fig. 6:**
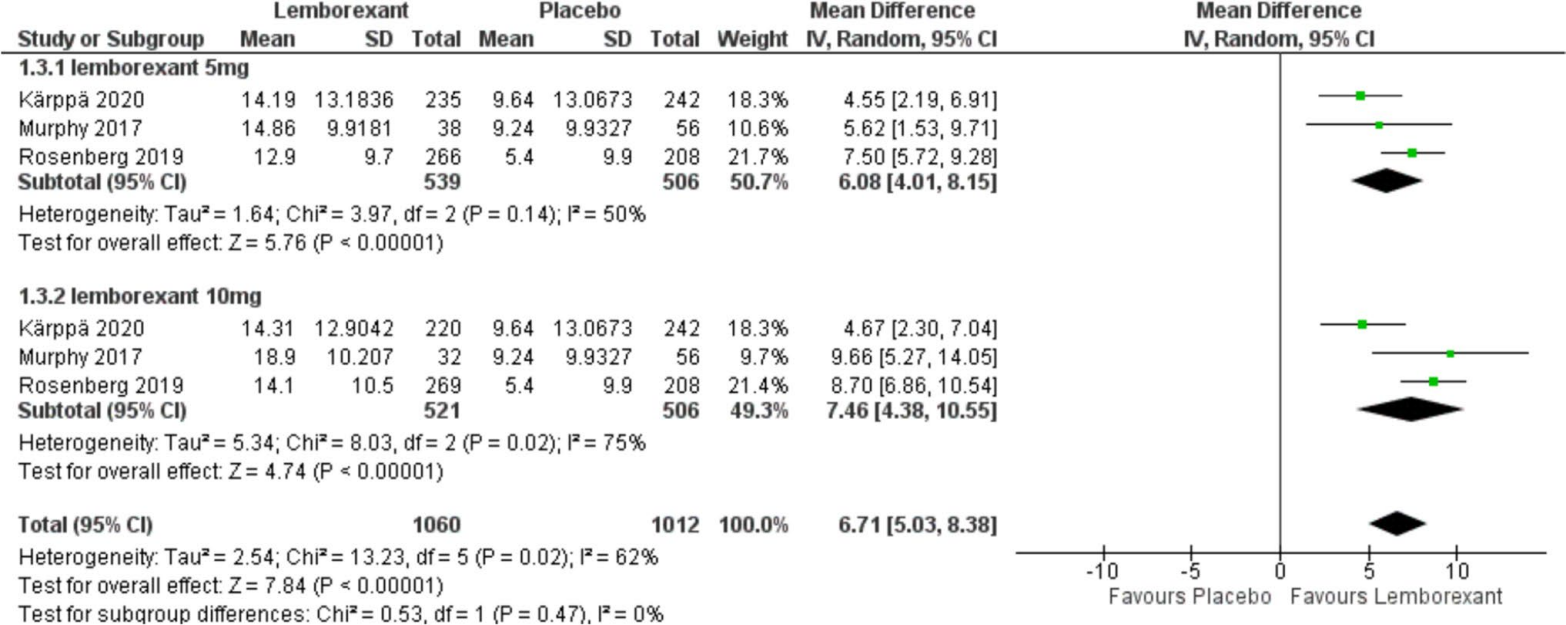
Forest plot showing the change in sleep efficiency from baseline

**Fig. 7:**
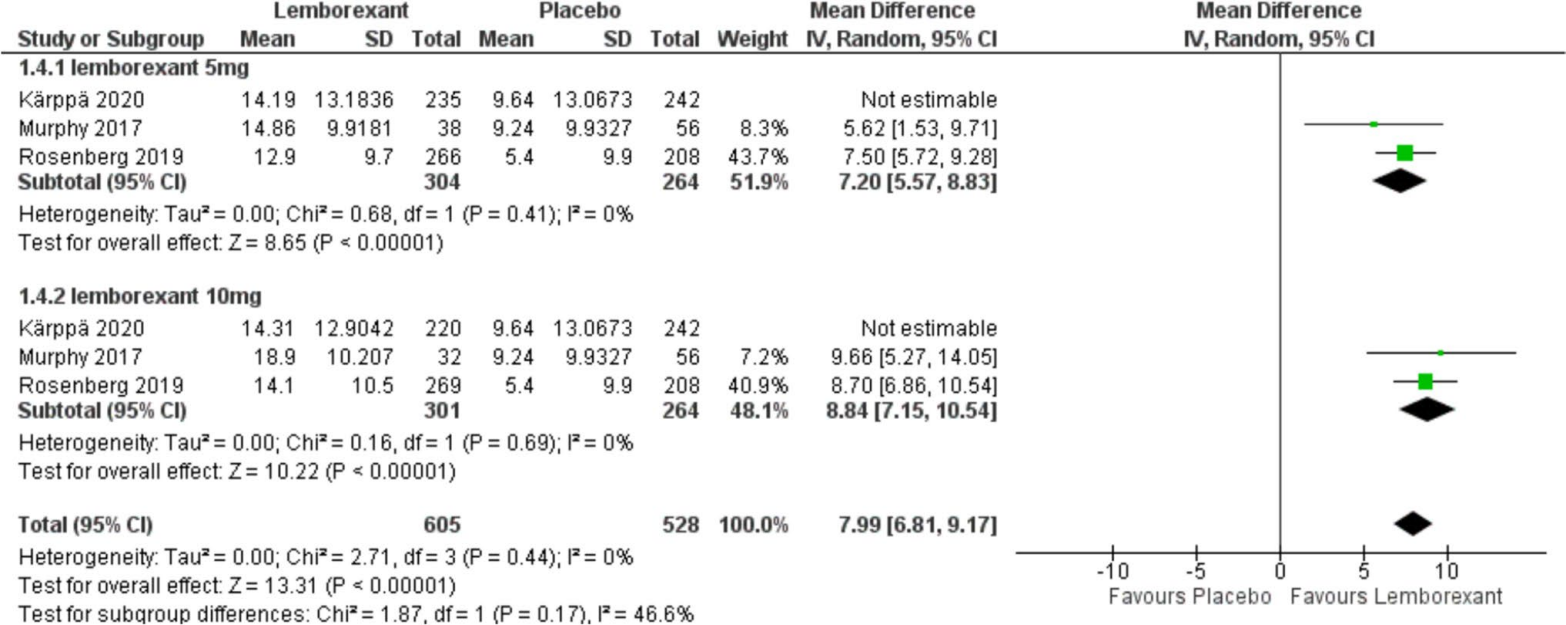
Forest plot showing sensitivity analysis for the change in sleep efficiency from baseline by removing Karppa 2020

The change in sleep efficiency from baseline was assessed in three of included studies (Murphy et al. 2017; Rosenberg et al. 2019; Kärppä et al. 2020) and calculated as following (sSE; [subjective total sleep time/subjective time in bed] × 100%). Results showed that the overall MD between lemborexant and placebo favors lemborexant (MD = 6.08, 95% CI [4.01 to 8.15], *P* < 0.0001) and (MD = 7.46, 95% CI [4.38 to 10.55], *P* < 0.0001) with 5 mg and 10 mg simultaneously. There was no statistically significant difference between the two groups (*P* = 0.47). Due to the marked heterogeneity with both 5 mg (*P* = 0.14, *I*^2^ = 50%) and 10 mg (*P* = 0.002, *I*^2^ = 75%), sensitivity analysis was performed for both outcomes. After removing Karppa 2020, pooled studies were homogenous in both doses (*I*^2^ = 0%) (*I*^2^ = 48%) (Figs. 6 and 7).

**Fig. 8:**
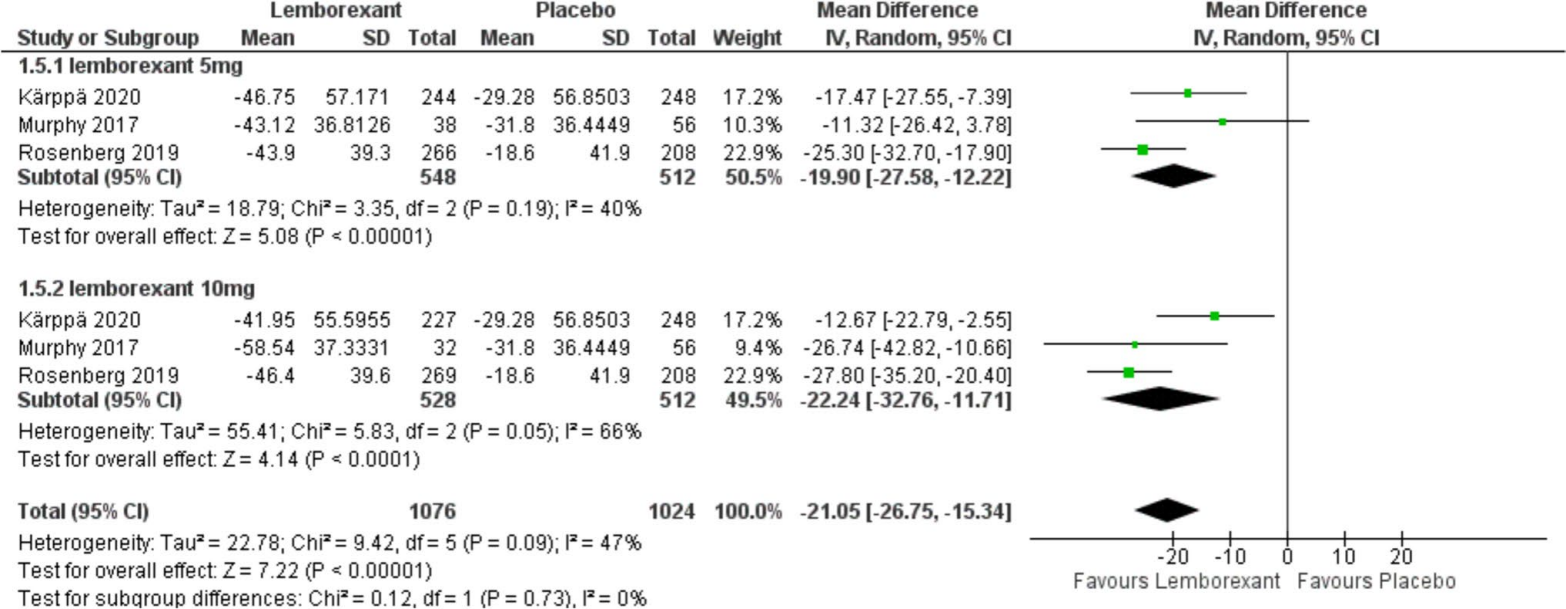
Forest plot showing the change in WASO from baseline

**Fig. 9:**
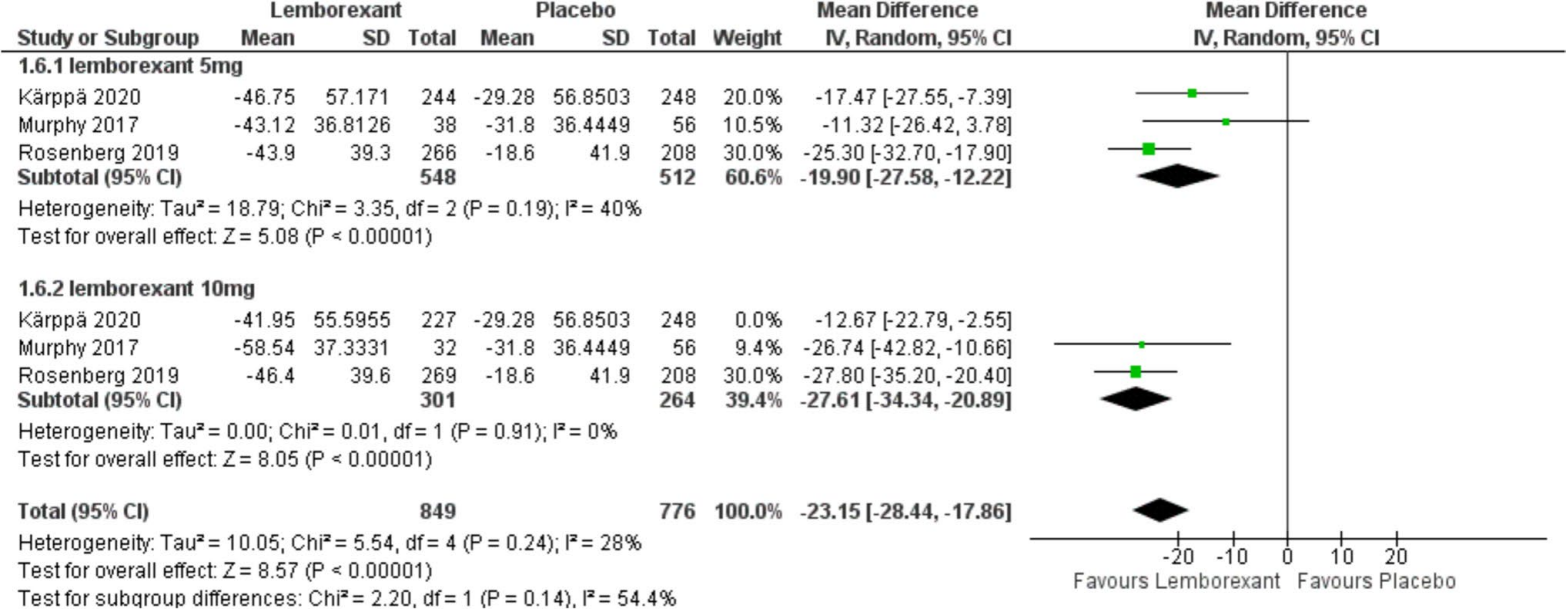
Forest plot showing sensitivity analysis for the change in WASO from baseline by removing Karppa 2020

The change in WASO from baseline was assessed in three of included studies (Murphy et al. 2017; Rosenberg et al. 2019; Kärppä et al. 2020) and defined as the sum of estimated minutes of wake after initial sleep onset until the participant got out of bed for the day. Analysis showed that lemborexant significantly reduces WASO compared to placebo (MD =  − 19.9, 95% CI [− 27.58 to − 12.22], *P* < 0.0001) and (MD =  − 22.24, 95% CI [− 32.76 to − 11.71], *P* < 0.0001) with 5 mg and 10 mg simultaneously. There was no statistically significant difference between the two groups (*P* = 0.73). Due to the marked heterogeneity with 10 mg (*P* = 0.05, *I*^2^ = 66%), sensitivity analysis was performed. After removing Karppa 2020, pooled studies were homogenous (*I*^2^ = 0%) (Figs. 8 and 9).

### Safety outcomes

Concerning treatment emerging adverse effects (TEAEs), serious TEAEs, TEAEs causing permanent discontinuation of study drug, and headache they did not show any statistically significant difference between lemborexant and placebo; while treatment-related TEAEs (RR = 1.94, 95% CI [1.61 to 2.33], *P* < 0.0001) and somnolence (RR = 4.95, 95% CI [3.08 to 7.96], *P* < 0.0001) favored placebo group. Additionally, they all were homogenous except TEAEs which showed heterogeneity (*I*^2^ = 68%). The heterogeneity was resolved by removing Karppa 2020.

A summary of the safety outcomes measures is found in Table 3 and the figures of the forest plots in the supplementary file (Figure S1-6).

**Table 3:** Safety outcomes analysis

Adverse event	No. of studies	No. of participants	RR [95% CI]	*P*-value	Heterogeneity
TEAEs (5 mg)	4	1338	1.00 [0.90, 1.12]	*P* = 0.98	Chi^2^ = 1.94, (*P* = 0.59); *I*^2^ = 0%
TEAEs (10 mg)	4	1339	1.22 [0.89, 1.67]	*P* = 0.21	Chi^2^ = 9.42, (*P* = 0.02); *I*^2^ = 68%
Serious TEAEs (5 mg)	3	1202	1.43 [0.52, 3.92]	*P* = 0.49	Chi^2^ = 0.87, (*P* = 0.65); *I*^2^ = 0%
Serious TEAEs (10 mg)	2	1250	1.62 [0.58, 4.51]	*P* = 0.36	Chi^2^ = 0.46, (*P* = 0.5); *I*^2^ = 10%
TEAEs leading to study withdrawal (5 mg)	2	1110	1.05 [0.51, 2.15]	*P* = 0.89	Chi^2^ = 0.1, (*P* = 0.75); *I*^2^ = 0%
TEAEs leading to study withdrawal (10 mg)	2	1112	2.04 [1.09, 3.80]	*P* = 0.03	Chi^2^ = 0.43, (*P* = 0.51); *I*^2^ = 0%
Headache (5 mg)	3	1202	1.24 [0.82, 1.87]	*P* = 0.31	Chi^2^ = 0.43, (*P* = 0.81); *I*^2^ = 0%
Headache (10 mg)	3	1198	0.97 [0.62, 1.51]	*P* = 0.89	Chi^2^ = 0.92, (*P* = 0.63); *I*^2^ = 0%
Somnolence (5 mg)	4	1338	3.84 [1.93, 7.62]	*P* < 0.001	Chi^2^ = 1.75, (*P* = 0.63); *I*^2^ = 0%
Somnolence (10 mg)	4	1339	6.26 [3.24, 12.07]	*P* < 0.001	Chi^2^ = 1.7, (*P* = 0.64); *I*^2^ = 0%
Treatment-related TEAE (5 mg)	4	1338	1.73 [1.33, 2.27]	*P* < 0.001	Chi^2^ = 0.57, (*P* = 0.9); *I*^2^ = 0%
Treatment-related TEAE (10 mg)	4	1339	2.15 [1.66, 2.78]	*P* < 0.001	Chi^2^ = 1.16, (*P* = 0.76); *I*^2^ = 0%

### Leave one out (sensitivity) and subgroup analysis

Sensitivity analysis was conducted on all efficacy outcomes by omitting one study each time to evaluate the robustness of the results. All outcomes were robust, and the results did not change by removing any of the included studies except sleep onset latency which showed insignificant results in multiple scenarios. A subgroup analysis was conducted to all outcomes based on different doses (5 mg and 10 mg) (Table S1). Results of the subgroup analysis revealed that there was no statistical difference between 5 and 10 mg in all outcomes.

### Meta-regression analysis

A meta-regression was performed to evaluate whether the effect estimates of the efficacy outcomes affected by their baseline and age or not. By using age and baseline values of each outcome as predictors, it was found that the effect of lemborexant was not dependent on these variables (Tables S2–4).

## Discussion

### Background

Orexin is a neuropeptide generated in the hypothalamus that regulates the sleep–wake cycle. It enhances wakefulness and arousal while also regulating appetite. Orexin is crucial for keeping the body awake and alert throughout the day (Toor et al. 2021). The brain contains two types of orexin receptors: OX1R and OX2R. Lemborexant is a dual orexin receptor antagonist with high selectivity to OX1R and OX2R works by competitively blocking these receptors (Mieda 2017). This causes the inhibition of orexin’s action owing to the decreased signals of wakefulness, thus promoting sleep (Zammit and Krystal 2021). This is why orexin antagonists belong among the new treatment possibilities of insomnia since they address the problem in starting and maintaining sleep without disturbing sleep architecture.

### Main findings

The present systematic review and meta-analysis examined the use of lemborexant in managing insomnia with special attention to measuring sleep onset latency, period of awake after sleep onset and sleep efficiency, and reviewing the safety of this therapy.

Sleep onset latency (SOL) assesses the period at the beginning of the sleep cycle, specifically the time it takes for a person to transition from full wakefulness to the first stage of non-REM (NREM) sleep. Findings of meta-analysis revealed that lemborexant 5-mg and 10-mg doses were effective in reducing SOL period as compared to placebo with 10-mg dose achieving higher reduction in SOL. Furthermore, sensitivity analysis was performed to address heterogeneity in this outcome, leaving out the Mayleben 2021 trial, which differed from the other trials included in the analysis in that this crossover study used an objective rather than subjective method of assessing the Sleep onset latency period. However, this sensitivity analysis had no effect on the overall effect estimate, which favored lemborexant at both doses. This suggests that lemborexant has a clear impact on a primary symptom of insomnia: difficulty initiating sleep.

Wake After Sleep Onset (WASO) quantifies the time during sleep maintenance defined as “the amount of time that one is awake prior to final arising, following the initial sleep episode.” It measures the breaks in sleep continuity, which take place after the sleeper has gone into the sleep cycle, generally during the non-REM and REM stages of the sleep cycle. As such, the result of this meta-analysis noted both doses of lemborexant, 5 mg and 10 mg, resulted in significant decrease of WASO either way but better in the 10-mg group. This means that Lemborexant not only helps the patients in falling asleep but also maintains sleep and adds more to the total duration of sleep. As with the other outcomes, heterogeneity was initially present but was effectively addressed through sensitivity analysis, further reinforcing the consistency of lemborexant’s effects on sleep maintenance. This solid evidence consolidates that lemborexant could effectively improve a crucial insomnia complaint, maintenance of sleep.

Lemborexant also achieved favorable results in sleep efficiency, a classical metric which indicates how long one remains asleep whilst in bed, as well. Both doses of lemborexant improved sleep efficiency in comparison to placebo revealing that the drug’s function extends to assuring that those who use it not only fall asleep quicker but also remain asleep for longer. This is critical since insomniacs are usually afflicted with sleep fragmentation and therefore their sleep quality suffers. The initial heterogeneity that was noted in the studies above was rooted in the omission of a specific study and the conclusiveness of the results is thereby enhanced. This finding supports the conclusion that the use of lemborexant will promote sleep that is undisturbed thereby raising the quality of sleep and the benefits of such to patients suffering from insomnia.

In terms of safety, both doses showed restively good safety profile as they are safe and well tolerated with no Treatment emerged adverse events, serious adverse events or headaches. However, meta-analysis showed that lemborexant showed higher risk of drug related adverse events and adverse events led to drug discontinuation. Mostly this adverse event is somnolence. Both doses showed that somnolence was more frequent in patients taking lemborexant compared to placebo. Somnolence, or excessive daytime sleepiness, is a known side effect of many sleep medications and could reflect lemborexant’s potent effect on sleep processes.

Overall, the meta-analysis showed that lemborexant is an efficient and rather safe treatment for the improvement of various sleep aspects in patients suffering from insomnia with a stable performance profile across various doses. It is an attractive therapeutic option as it improves sleep onset latency, efficiency of sleep, and WASO but great caution should be practiced when this treatment is prescribed owing to some adverse effects.

### Clinical implications

The results of this systematic review and meta-analysis demonstrate that lemborexant is a useful measure for the treatment of insomnia, especially with respect to sleep onset latency (SOL), wake after sleep onset (WASO), and sleep efficiency. Both 5-mg and 10-mg doses of lemborexant decreased the amount of time it took to fall asleep, and the time patients spent awake at night, thus ensuring improved sleep continuity. The drug also improved sleep efficiency, meaning that patients had more of their time in bed dedicated to actual sleep which is important for enhancing sleep quality. The use of lemborexant in most cases was safe and well tolerated; however, towards the downside, mild and excessive daytime sleepiness may be encountered particularly at high doses. All results obtained from this study confirm that lemborexant is effective in the treatment of insomnia, enhancing initiation and maintenance of sleep, although clinicians should be careful of the risk of sedation in susceptible patients.

### Previous research

Kishi et al. (Kishi et al. 2020) in their systematic review and meta-analysis compared lemborexant to suvorexant and placebo and found a favorable effect of lemborexant 10 mg over suvorexant and placebo however this evidence was inconclusive due to limited number of included studies, only two clinical trials for lemborexant. Moreover, analysis of safety data was deficient.

Crescenzo and colleagues (Crescenzo et al. 2022) conducted a network meta-analysis on all pharmacological treatments for insomnia and concluded that lemborexant side to side with eszopiclone are ranked better with favorable profiles for insomnia. However, safety data of lemborexant was inconclusive and all outcomes assessing all aspects of various insomnia symptoms were missed.

Similarly, Rocha and colleagues (Rocha et al. 2023) conducted a meta-analysis pooling treatment effect of all orexin receptor antagonists and concluded the same findings that OR antagonists could effectively improve insomnia symptoms. However, no safety data were provided. In addition, the study lacked a comprehensive good number of clinical trials assessing the efficacy of lemborexant. Ultimately, no conclusions can be drawn specifically about lemborexant, as the analysis encompassed all orexin receptor antagonists.

Overall, previous systematic review and meta-analyses did not provide conclusive evidence about the efficacy and safety of lemborexant on insomnia due to limited number of clinical trials and lack of adequate outcomes to assess lemborexant effect on all symptoms of insomnia.

Recent concerns regarding the safety of dual orexin receptor antagonists (DORAs), including lemborexant, have been highlighted in the systematic review and meta-analysis by Na et al. (2024), which assessed the clinical safety of FDA-approved DORAs with a focus on narcolepsy-like symptoms such as excessive daytime sleepiness (EDS), sleep paralysis, and hallucinations. Their findings align with our study’s safety analysis, which identified somnolence as the most prominent adverse effect of lemborexant. Na et al. demonstrated that DORAs significantly increased the risk of TEAEs, particularly EDS (RR = 3.48, 95% CI: 2.25–5.38, *P* < 0.001), while sleep paralysis and hallucinations, though rare, were observed. Additionally, they found that higher doses of DORAs were associated with a greater incidence of EDS, reinforcing our observation that lemborexant 10 mg may pose a higher risk of excessive sedation compared to the 5-mg dose (Na et al. 2024).

The network meta-analysis by Yue et al. (2023) provides a broad comparative assessment of the efficacy and tolerability of various pharmacological treatments for insomnia, including orexin receptor antagonists, benzodiazepine receptor agonists, and melatonin receptor agonists. Their findings indicate that orexin receptor antagonists, including lemborexant, are among the most effective options for improving sleep onset and maintenance while maintaining a favorable safety profile. Notably, lemborexant demonstrated comparable or superior efficacy to traditional benzodiazepine receptor agonists, with a lower risk of dependence and withdrawal symptoms. However, Yue et al. also reported that lemborexant was associated with a higher incidence of somnolence compared to melatonin receptor agonists and certain non-benzodiazepine hypnotics. This aligns with our findings, where lemborexant exhibited a significant risk of excessive daytime sleepiness (Yue et al. 2023).

Cost considerations are critical in determining the appropriateness of pharmacologic treatments for insomnia, particularly when benefits over placebo are modest. Based on recent U.S. pharmacy cost estimates, lemborexant (5 mg or 10 mg) costs approximately $4.50 per dose unit, while zolpidem (10 mg) is significantly cheaper at $0.68 per dose unit, and suvorexant (10 mg) is the most expensive at $15.97 per dose unit. Given this cost discrepancy, prescribing physicians must weigh the relative benefits and risks of lemborexant against lower-cost alternatives like zolpidem. Although lemborexant offers advantages such as a lower risk of dependency compared to benzodiazepine receptor agonists, its efficacy over zolpidem remains a key question (Rosenberg et al. 2019). In a head-to-head randomized controlled trial (Rosenberg et al. 2019), lemborexant was compared directly to zolpidem ER (6.25 mg) in older adults with insomnia and was found to provide greater improvements in sleep maintenance, though it showed no significant superiority in sleep onset latency reduction. Additionally, lemborexant was associated with a higher incidence of somnolence and treatment-emergent adverse events, suggesting that while it may benefit patients with sleep maintenance difficulties, its overall advantage over zolpidem is limited (Rosenberg et al. 2019).

### Strengths

This systematic review and meta-analysis has some strengths for a number of reasons. First, it represents a unique contribution to the evidence base by including four clinical trials, as opposed to previous analyses which were limited to two trials. Focusing on clinical phase studies ensures that only reliable and valid findings are produced. Second, the study outcomes broadly cover insomnia by evaluating its symptoms through all sleep cycle phases including sleep onset latency indirectly assessing initiation of sleep phase, wake after sleep onset indirectly assessing maintenance of sleep phase, and sleep efficiency or overall sleep quality. This detailed approach is valuable as it provides a thorough evaluation of the effect of lemborexant on insomnia. Furthermore, in comparison with evaluations by Kishi (Kishi et al. 2020), by Crescenzo (Crescenzo et al. 2022) or Rocha (Rocha et al. 2023) which had evaluative limitations on lemborexant safety, this review renders a definitive evaluative judgement on the safety of the drug. Hence the conclusions made here allow to enrich the understanding on lemborexant’s efficacy and safety and hence resolve up to the critical knowledge gaps within literature.

### Limitations and recommendations

It is important to acknowledge certain limitations when interpreting the findings. Firstly, our results are based on only four RCTs. Therefore, additional high-quality RCTs with larger sample sizes are necessary to confirm these results in the future. There were some estimates for incomplete data that were derived using statistical equations which certainly had a margin of error. Heterogeneity was present in some outcomes which may be due to the variation in the time of administrations, follow-up duration, sample size, population characteristics, and subjective evaluation in certain outcomes. However, these variations give the study results high generalizability and external validity. Additionally, studies over a longer timeframe are required to determine whether the superiority of lemborexant treatment remains consistent or shows further improvement over time. Considering the chronicity of insomnia, it is crucial to highlight the importance of assessing the long-term safety concerns such as the potential for addiction and rebound insomnia. In addition, it is essential to conduct head-to-head studies against active comparators such as Melatonin, Orexin, and Ambien to compare the efficacy and safety of lemborexant versus existing commercially available treatments. Finally, we were unable to assess publication bias due to the limited number of studies.

## Conclusion

To conclude, this systematic review and meta-analysis confirms that lemborexant can be considered a safe and effective option for the treatment and management of insomnia, particularly in the alleviation of SOL, WASO and sleep efficiency. Both doses administered in the structures, namely 5 mg and 10 mg, contributed significantly to the reduction of the time to sleep onset and also decomposition of sleep which translated into quality of sleep. Instances of somnolence related to other sleep drugs whereby it was possible to observe in few patients were high, though there was a general tolerance for lemborexant. The capacity of the drug to manage the major symptoms of insomnia would make the management very useful. In any case, with regards to lemborexant, it should be noted that some adverse events, particularly somnolence, need to be paid closer attention to by doctors. All in all, it offers considerable possibility of overcoming both the lack of sleep initiation and the lack of sleep duration in patients with insomnia.

## Supplementary Information

Below is the link to the electronic supplementary material.

Supplementary file1 (DOCX 124 KB)

## Data Availability

All source data for this work (or generated in this study) are available upon reasonable request.
