# Supplementary material for: Efficacy and safety of lemborexant vs placebo in treating adults with insomnia disorder: a systematic review and meta-analysis of 1976 patients
Source: Naunyn Schmiedebergs Arch Pharmacol. 2025 Apr 17;398(10):12911–26. doi: 10.1007/s00210-025-04072-4 (PMC12511177; doi:10.1007/s00210-025-04072-4)
Supplement: Supplementary file 1 — Supplementary file1 (DOCX 124 KB) [file 210_2025_4072_MOESM1_ESM.docx]

**Efficacy and Safety of Lemborexant vs Placebo in treating Adults with Insomnia disorder: A Systematic Review and Meta-Analysis of 1,976 Patients**

**Figure S1**. Analysis of TEAE risk ratio between Lemborexant and placebo.


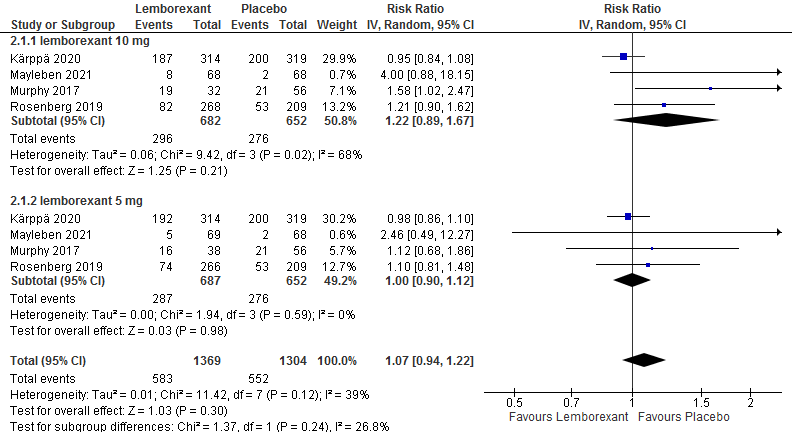


**Figure S2.** Analysis of treatment related AE risk ratio between Lemborexant and placebo.


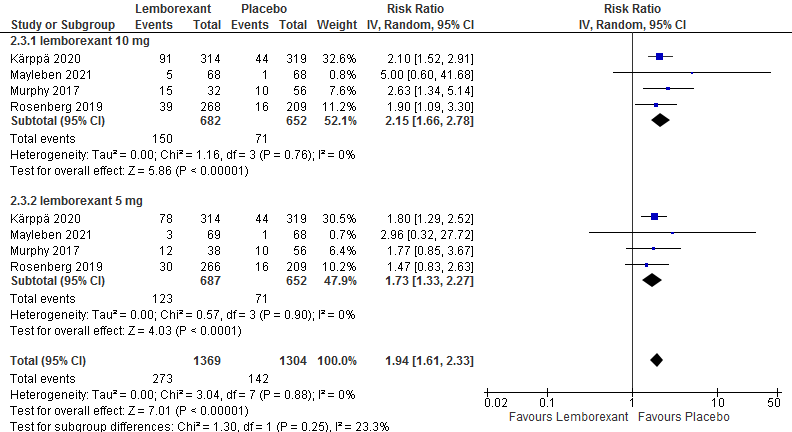


**Figure S3**. Analysis of TEAEs leading to discontinuation risk ratio between Lemborexant and placebo.


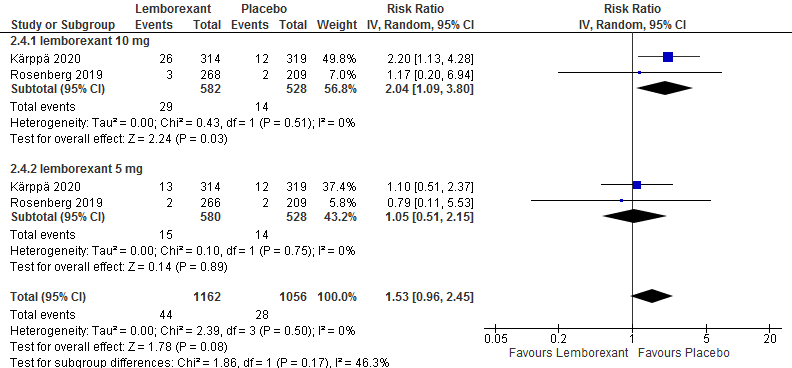


**Figure S4**. Analysis of Headche risk ratio between Lemborexant and placebo.


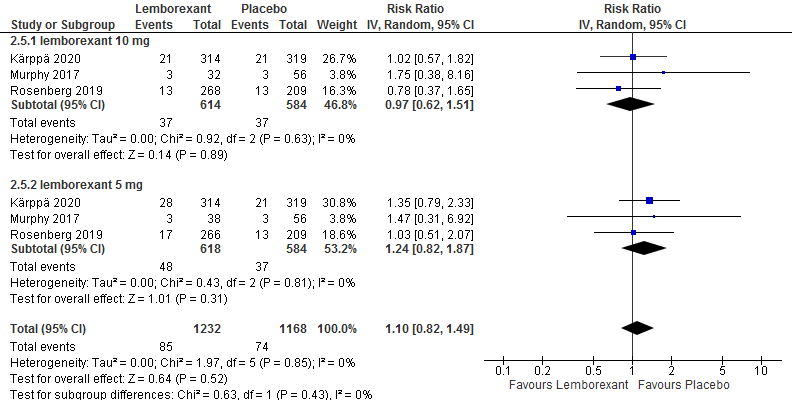


**Figure S5**. Analysis of Somnolence risk ratio between Lemborexant and placebo.


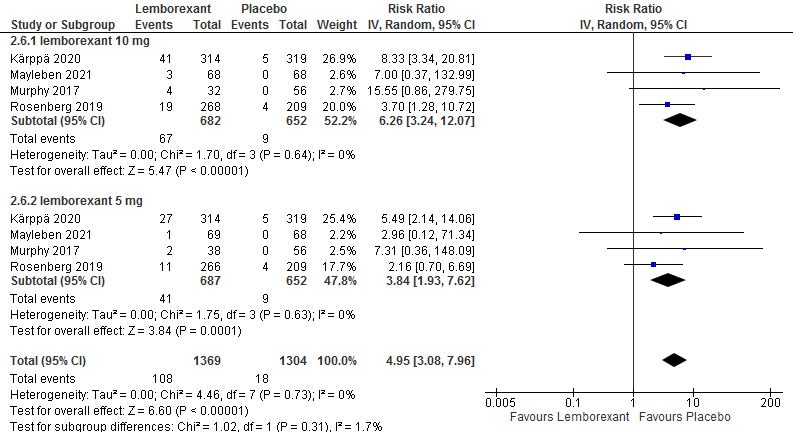


**Figure S6.** Analysis of serious AE risk ratio between Lemborexant and placebo.


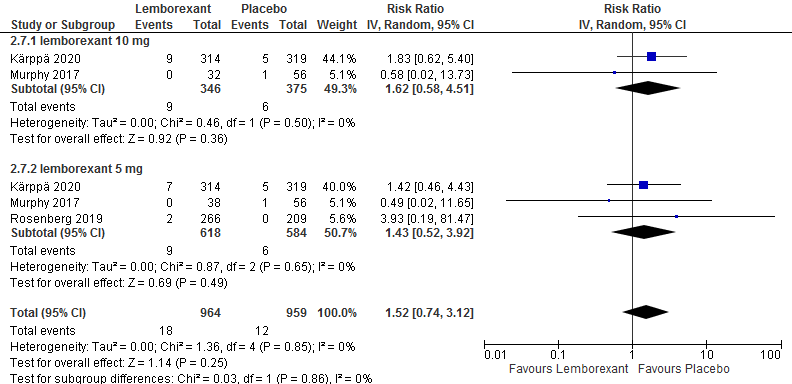


**Table. S1:** Leave one out analysis for the efficacy outcomes.

| **Study removed** | **No. of Participants** | **No. of trials** | **Quantitative data synthesis** | | | **Heterogeneity analysis** | |
| --- | --- | --- | --- | --- | --- | --- | --- |
|  |  |  | **MD** | **95% CI** | **P-value** | **P-value** | **I^2^(%)** |
| **Sleep onset latency (5 mg)** | | | | | | | |
| Kärppä 2020 | 705 | 2 | -8.80 [-18.47, 0.87] | | P= 0.07 | P<0.001 | 92% |
| Murphy 2017 | 1105 | 2 | -7.50 [-15.53, 0.53] | | P= 0.07 | P<0.001 | 93% |
| Rosenberg 2019 | 725 | 2 | -8.48 [-17.36, 0.39] | | P= 0.06 | P<0.001 | 94% |
| Mayleben 2021 | 1062 | 2 | -11.77 [-14.97, -8.56] | | P< 0.001 | P=0.61 | 0% |
| **Sleep onset latency (10 mg)** | | | | | | | |
| Kärppä 2020 | 705 | 2 | -11.52 [-21.50, -1.53] | | P= 0.02 | P<0.001 | 92% |
| Murphy 2017 | 1105 | 2 | -10.59 [-19.69, -1.49] | | P= 0.02 | P<0.001 | 94% |
| Rosenberg 2019 | 725 | 2 | -12.31 [-23.14, -1.48] | | P= 0.03 | P<0.001 | 94% |
| Mayleben 2021 | 1062 | 2 | -15.55 [-19.11, -11.98] | | P< 0.001 | P=0.54 | 0% |
| **Sleep efficiency (5 mg)** | | | | | | | |
| Kärppä 2020 | 568 | 2 | 7.20 [5.57, 8.83] | | P< 0.001 | P=0.41 | 0% |
| Murphy 2017 | 968 | 2 | 6.13 [3.25, 9.01] | | P< 0.001 | P=0.05 | 74% |
| Rosenberg 2019 | 588 | 2 | 4.82 [2.78, 6.86] | | P< 0.001 | P=0.66 | 0% |
| **Sleep efficiency (10 mg)** | | | | | | | |
| Kärppä 2020 | 568 | 2 | 8.84 [7.15, 10.54] | | P< 0.001 | P=0.69 | 0% |
| Murphy 2017 | 968 | 2 | 6.76 [2.81, 10.70] | | P< 0.001 | P=0.008 | 86% |
| Rosenberg 2019 | 588 | 2 | 6.81 [1.97, 11.65] | | P= 0.006 | P=0.05 | 74% |
| **Wake after sleep onset (5 mg)** | | | | | | | |
| Kärppä 2020 | 568 | 2 | -19.92 [-33.25, -6.59] | | P= 0.003 | P=0.1 | 62% |
| Murphy 2017 | 968 | 2 | -22.16 [-29.68, -14.64] | | P< 0.001 | P=0.22 | 34% |
| Rosenberg 2019 | 588 | 2 | -15.58 [-23.96, -7.19] | | P< 0.001 | P=0.51 | 0% |
| **Wake after sleep onset (10 mg)** | | | | | | | |
| Kärppä 2020 | 568 | 2 | -27.61 [-34.34, -20.89] | | P< 0.001 | P=0.91 | 0% |
| Murphy 2017 | 968 | 2 | -20.64 [-35.45, -5.84] | | P= 0.006 | P=0.02 | 82% |
| Rosenberg 2019 | 588 | 2 | -18.26 [-31.76, -4.77] | | P= 0.008 | P=0.15 | 53% |

**Table. S2:** Meta regression of sleep efficiency outcome on age and its value at baseline.

| Model Coefficients - Sleep efficiency | | | | | | | | | |
| --- | --- | --- | --- | --- | --- | --- | --- | --- | --- |
|  |  |  |  |  |  |  |  |  |  |
| **Predictor** | | **Estimate** | | **SE** | | **t** | | **p** | |
| Intercept |  | 8.377 |  | 15.3066 |  | 0.547 |  | 0.622 |  |
| Age |  | -0.297 |  | 0.0968 |  | -3.065 |  | 0.055 |  |
| Sleep efficiency at baseline |  | 0.351 |  | 0.2589 |  | 1.357 |  | 0.268 |  |
|  | | | | | | | | | |

**Table. S3:** Meta regression of sleep onset latency outcome on age and its value at baseline.

| Model Coefficients – Sleep onset latency | | | | | | | | | |
| --- | --- | --- | --- | --- | --- | --- | --- | --- | --- |
|  |  |  |  |  |  |  |  |  |  |
| **Predictor** | | **Estimate** | | **SE** | | **t** | | **p** | |
| Intercept |  | -5.423 |  | 87.238 |  | -0.0622 |  | 0.953 |  |
| Sleep onset latency Baseline |  | -0.130 |  | 0.916 |  | -0.1417 |  | 0.893 |  |
| Age |  | -0.160 |  | 0.902 |  | -0.1776 |  | 0.866 |  |

**Table. S4:** Meta regression of WASO outcome on age and its value at baseline.

| Model Coefficients – WASO | | | | | | | | | |
| --- | --- | --- | --- | --- | --- | --- | --- | --- | --- |
|  |  |  |  |  |  |  |  |  |  |
| **Predictor** | | **Estimate** | | **SE** | | **t** | | **P** | |
| Intercept |  | -84.825 |  | 32.558 |  | -2.605 |  | 0.080 |  |
| Age |  | 0.429 |  | 0.419 |  | 1.023 |  | 0.381 |  |
| WASO baseline |  | 0.119 |  | 0.211 |  | 0.564 |  | 0.612 |  |
